# Formation of amorphous calcium carbonate in caves and its implications for speleothem research

**DOI:** 10.1038/srep39602

**Published:** 2016-12-22

**Authors:** Attila Demény, Péter Németh, György Czuppon, Szabolcs Leél-Őssy, Máté Szabó, Katalin Judik, Tibor Németh, József Stieber

**Affiliations:** 1 Institute for Geological and Geochemical Research, RCAES, Hungarian Academy of Sciences, Budaörsi út 45, Budapest, H-1112, Hungary; 2 Institute of Materials and Environmental Chemistry, Research Centre for Natural Sciences, Hungarian Academy of Sciences, Magyar tudósok körútja 2., Budapest, H- 1117, Hungary; 3 Department of Physical and Applied Geology, Eötvös Loránd University, Pázmány Péter sétány. 1/C, Budapest, H-1117, Hungary; 4 Stieber Environmental Ltd., Nyerges u. 6., Budapest, H-1181, Hungary

## Abstract

Speleothem deposits are among the most valuable continental formations in paleoclimate research, as they can be dated using absolute dating methods, and they also provide valuable climate proxies. However, alteration processes such as post-depositional mineralogical transformations can significantly influence the paleoclimatic application of their geochemical data. An innovative sampling and measurement protocol combined with scanning and transmission electron microscopy, X-ray diffraction and Fourier transform infrared spectroscopy is presented, demonstrating that carbonate precipitating from drip water in caves at ~10 °C contains amorphous calcium carbonate (ACC) that later transforms to nanocrystalline calcite. Stable oxygen isotope fractionations among calcite, ACC and water were also determined, proving that ACC is ^18^O-depleted (by >2.4 ± 0.8‰) relative to calcite. This, in turn, has serious consequences for speleothem-based fluid inclusion research as closed system transformation of ACC to calcite may induce a negative oxygen isotope shift in fluid inclusion water, resulting in deterioration of the original compositions. ACC formation increases the speleothems’ sensitivity to alteration as its interaction with external solutions may result in the partial loss of original proxy signals. Mineralogical analysis of freshly precipitating carbonate at the studied speleothem site is suggested in order to determine the potential influence of ACC formation.

Speleothems (cave-hosted carbonate deposits) are among the most important climate archives, as their formation age, texture and geochemical composition provide information on temperature, amount of precipitation, and even seasonality, i.e. changes in the relative amount of precipitation during cold and warm seasons^1^. The preservation of the original climate-related signal (“proxy”) is therefore of crucial importance. The recrystallization and alteration of the original carbonate may significantly influence the commonly used U-Th age dating method, stable C and O isotope composition, as well as the distribution and isotope ratios of trace elements^2^,^3^,^4^,^5^.

A recent study^6^ has shown evidence for recrystallization-induced alteration processes that affected stable isotope compositions of inclusion-hosted water trapped in stalagmites. These were tentatively attributed to the formation of amorphous calcium carbonate (ACC) and its transformation to calcite. Potential effects of vaterite and aragonite formation has also been discussed and excluded by Demény *et al*.^6^ on the basis of available carbonate-water oxygen isotope fractionation relationships. Additionally, vaterite and aragonite has not been detected in the Baradla cave system, in spite of an extensive survey on different carbonate deposit types (freshly precipitated carbonate, stalagmites, flowstones, this study and Demény *et al*.^6^). As detected by the earlier study^6^, the stable hydrogen and oxygen isotope compositions (expressed as δD and δ^18^O values) of water trapped in the fluid inclusions of selected speleothems of the Baradla cave system shows that, whereas hydrogen isotope values reflect the composition of the cave drip water from which the carbonate precipitated, the oxygen isotope data of the inclusion-hosted water display increasingly negative shifts. The observed negative δ^18^O(water) shift starts from the stalagmite’s surface and increase inwards, towards the older layers. Although Demény *et al*.^6^ did not provide direct evidence for the presence of ACC in the stalagmites or on their surface, they did report the presence of nanocrystalline (<50 nm) calcite, from which ACC in the role of a precursor material may be inferred. According to experimental and empirical data, O isotope fractionation between ACC and water is smaller than that for crystalline carbonate^7^,^8^,^9^. Calcite crystallization could be responsible for driving the inclusion-hosted water in a negative direction, enabling it to reach the larger fractionation value, and thus could provide explanation for the observed negative δ^18^O shift^6^.

Detection of ACC is rather difficult in cave deposits, as ACC can undergo transformation to calcite in minutes in a hydrous environment, and even stabilizing compounds like Mg or organic matter^10^,^11^ are only capable of extending its stability to some weeks. Taking into consideration the general precipitation rate (0.1 to 1 mm per year), the collection of carbonate in appropriate amounts for mineralogical or geochemical analyses requires several months. Over the course of such a long collection time, however, the original ACC can be transformed into calcite. Although ACC preparation in the laboratory is a routine procedure^12^,^13^,^14^, its synthesis requires conditions distinctly different from those to be found in natural cave environments, e.g. mixing of CaCl_2_ and NaCO_3_^12^,^13^ or (NH_4_)_2_CO_3_^14^ solutions. Hence, the preparation conditions and characteristics of synthetic ACC render it inappropriate to function as an analogue of its natural counterpart, thus it cannot provide the information sought.

In the present paper, the issue of ACC *vs.* calcite precipitation was approached via the application of an innovative sample collection method in which transmission electron microscopy (TEM) grids and silica wool are placed directly under the dripping water in the studied caves. These sample holders were able to collect enough carbonate precipitate for mineralogical and isotopic analyses in relatively short times (24 hours and 2 weeks, respectively). This had the effect of allowing direct analyses without further sample concentration. Evidence is presented for ACC formation in natural cave environments and an estimate for calcite-ACC oxygen isotope fractionation at the studied cave temperature (10 ± 0.5 °C, the average temperature and its scatter of several sites in two caves) is provided.

## Results and Discussion

**Figure 1:**
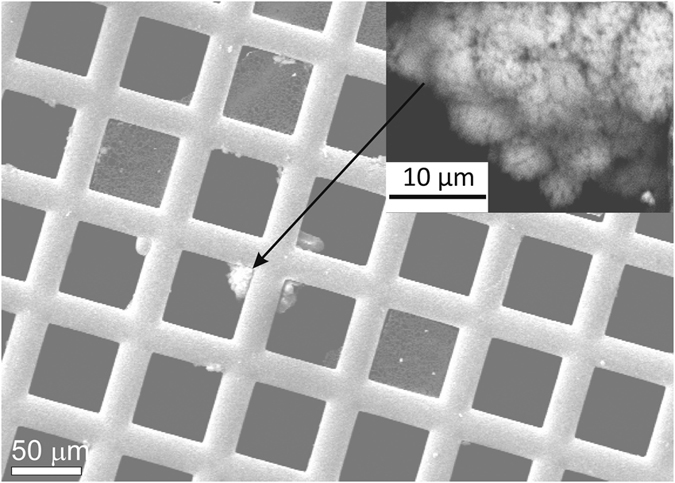
SEM image of carbonate precipitate on a Cu TEM grid (sample grid #2, Baradla). Globular forms are attached to the Cu grid. The insert (rotated relative to the main image) shows a globular CaCO_3_ aggregate. The sample was not covered by carbon or metal in order to save it for further analyses.

The temperature, conductivity, pH values and stable isotope compositions of the drip water were recorded during sampling, as was the CO_2_ content of the cave air. These are listed in Table 1. Grids and silica wool samples were analysed for several sampling periods from the Baradla and the Pál-völgyi caves, but due to the very small amount of deposited material, only some of the grids yielded enough sample material to allow their study using a combination of methods. Grids that were placed under the drip waters for 24 hours could only be analysed using TEM, whereas the precipites collected for ~2 weeks could be studied employing a range of methods. Most of the grids showed traces of mainly clay minerals and idiomorphic calcite. However, CaCO_3_ was occasionally deposited in globular forms (Fig. 1) or thin films over lacey carbon (see later).

### Fresh carbonate precipitate

**Figure 2:**
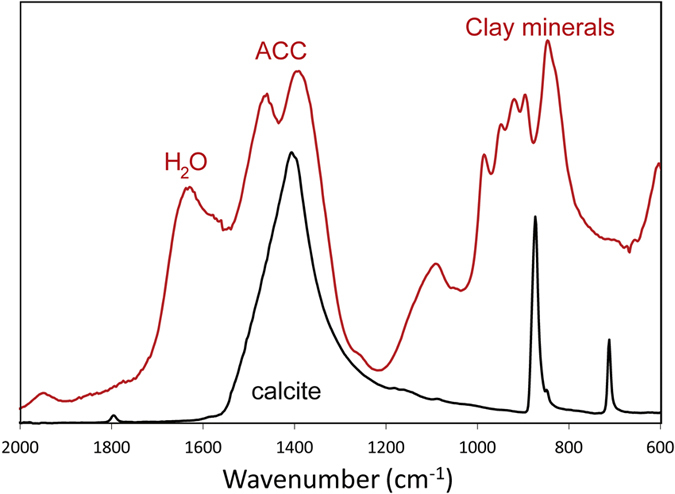
FTIR spectra of a carbonate globule from sample grid #2 (red line) and the surface carbonate of a stalagmite (sample NU-2, black line) of the Baradla Cave. Besides the characteristic split peak at 1393 and 1460 cm^−1^, attributed to ACC, peaks corresponding to molecular water^15^ and clay minerals^16^ are also shown. Note that the calcite peaks at 712 and 874 cm^−1^ are missing from the spectrum of the carbonate globule from the grid sample.

FTIR spectroscopy, micro-focus XRD and TEM were used in order to characterize the structure of the carbonate precipitate. The FTIR spectra (Fig. 2) obtained for one of the grids (sample grid #2) were similar to that of the experimentally prepared ACC^14^. The splitting of peaks at 1393 and 1460 cm^−1^ wave numbers was observed, matching the data in the literature reported for ACC^15^ (1384 and 1472 cm^−1^). The wide peak centered on 1635 cm^−1^ indicates molecular water^15^, and the series of peaks between 800 and 1200 cm^−1^ is consistent with spectra obtained for clay minerals^16^.

**Figure 3:**
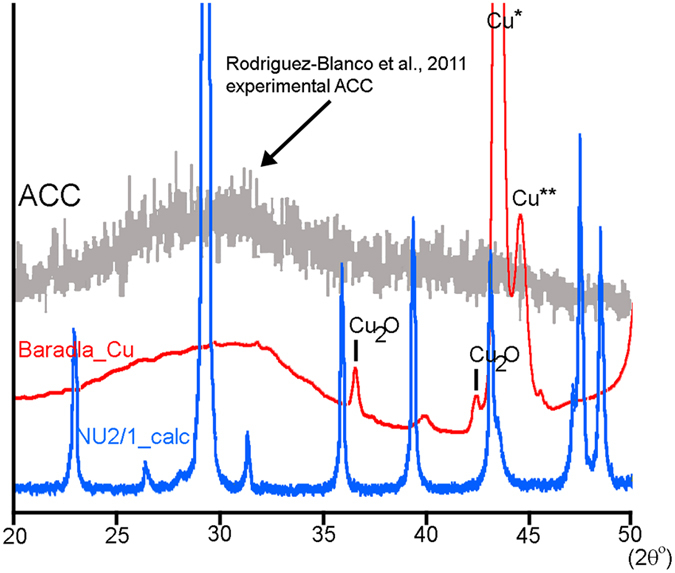
XRD pattern (red) of the material precipitated on the lacey carbon Cu TEM grid shows evidence for ACC. The precipitate was collected for 20 hours from dripping water. The blue pattern (NU2/1_calc) measured from the youngest layer of the stalagmite NU-2 (collected at the site of the experiments) corresponds to calcite. Only a broad hump (indicated by the black arrow) between 25° and 35° 2Θ occurs for the red pattern, and this feature is the same as that of ACC (grey pattern)^11^. The strongest peak (Cu*) of the sample Baradla_Cu corresponds to Cu(111). A Cu** peak arises from the Cu(Kα2) contribution. The black lines at 36.48° and 42.37° 2Θ indicate Cu_2_O peaks.

The same grid that yielded the characteristic ACC FTIR spectrum was also analysed by means of micro-focus XRD. A 2Θ range of 20 to 50° was selected because this range is uniquely capable of defining ACC^13^. The sample showed no traces of calcite, but a broad hump with a maximum at ~31.6° 2Θ (Fig. 3), matching both the shape and the maximum of experimentally precipitated ACC^11^,^13^.

**Figure 4:**
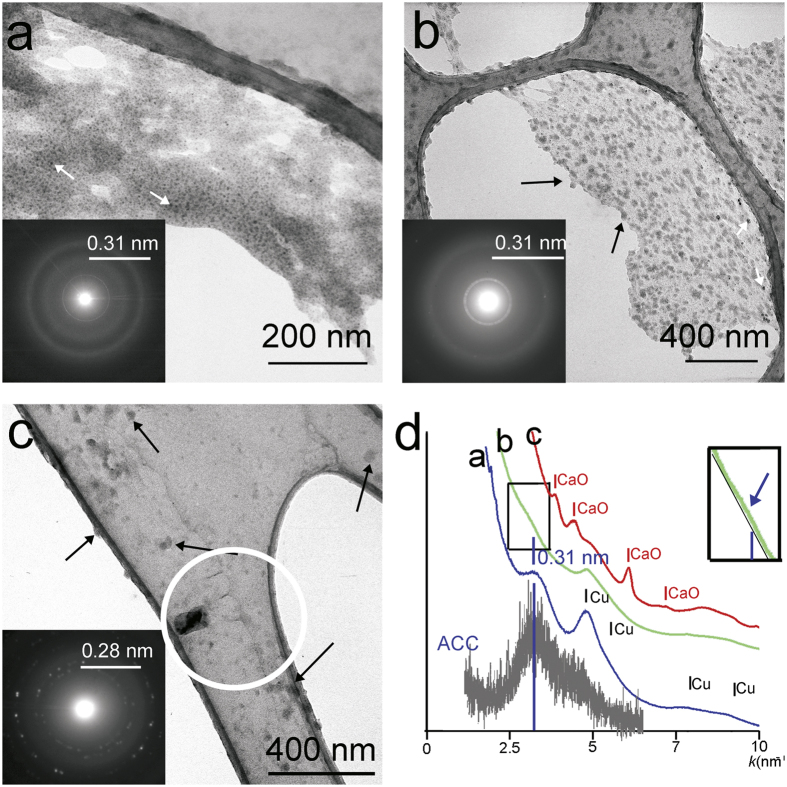
TEM images and diffraction features of the carbonate material precipitated on the lacey carbon surface of the Cu grid. The precipitates occur as thin films in (**a**) and (**b**) and as rounded particles in (**c**). The carbonate film is hollow, containing Cu crystals (white arrows) 2–10 nm in size and has a ragged edge. Rounded clusters (black arrows) 20–30 nm in size are also apparent in (**b**). The particles in (**c**) are 20–40 nm in size (black arrows). Similar particles have been reported in previous TEM investigations and interpreted as ACC^17^. Radial distributions (plotted in (**d**)) generated from the SAED intensities of (**a**) and (**b**) display a broad hump, with diffraction maxima at 0.31 nm (blue line). This feature is consistent with the diffraction characteristic of synthetic ACC (grey pattern)^11^ (X-ray data was converted to scattering values (k) for comparison). The hump is well separated from the background in distribution a, whereas it is poorly separated in distribution (**b**). In order to enhance the visibility of the separation of distribution b we included an inset (**d**). The separation of the hump is likely to correspond to a different carbonate cluster ordering. The black lines in (**d**) indicate Cu peaks. The SAED pattern from the white circle region in (**c**) shows discrete reflections and the radial distribution generated from the pattern displays the characteristic peaks of CaO. This product most likely resulted from the Ca-carbonate and electron beam interaction.

TEM analysis of the fresh precipitates showed either a thin (10–40 nm), commonly hollow, CaCO_3_ film consisting of 10–30 nm size rounded clusters (Fig. 4a,b) or 20–40 nm size particles (Fig. 4c), similar to published ACC TEM images^17^. The clusters contain <5 nm Cu nanocrystals, which probably originated from the alteration of the copper grid. The SAED patterns of the CaCO_3_ films showed poorly resolved diffuse rings, consistent with the diffraction characteristic of ACC. The SAED pattern of the small particles indicated the presence of CaO (lime). Lime may act as a proxy for ACC as interaction between the electron beam and ACC may release CO_2_, resulting in the formation of lime^17^. The upper particle size of the fresh precipitate matches with the average size (40 nm) of the calcite nanocrystals reported in the outermost layer of recently forming stalagmites^6^.

### Carbonate transformation

**Figure 5:**
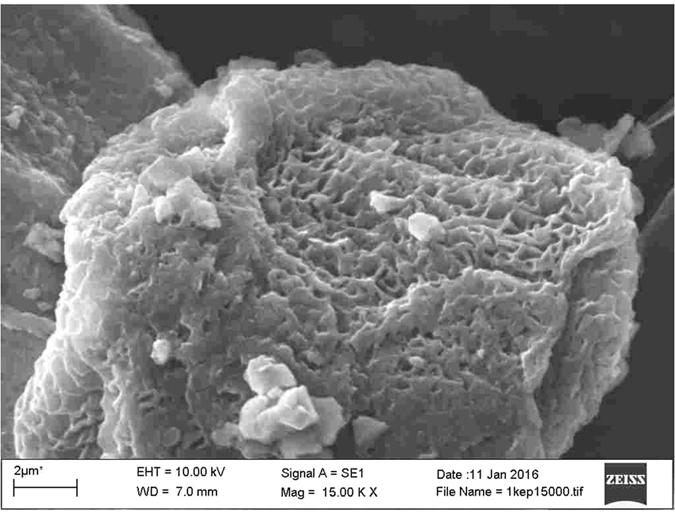
SEM image of a porous calcite grain formed on a CU TEM grid after three weeks storage. It is important to note that no globular CaCO_3_ forms were detected after the long storage.

SEM pictures were taken of grids immediately after collection and again with the same samples after about 3 weeks’ storage in a refrigerator. The analysis of the fresh samples revealed the formation of globular CaCO_3_, while this form had disappeared after 3 weeks, being replaced by porous, crystalline carbonate (Fig. 5).

FTIR analyses of the stored grid with recrystallized carbonate match with that of calcite, no ACC-like spectra were observed. The formation of porous calcite is in accordance with the transformation of ACC to calcite. Since the ACC contains up to one H_2_O molecule per formula unit^14^, and the molar volume of ACC (73 cm^−3^ mol^−1^) exceeds the sum of molar volumes of calcite (36 cm^−3^ mol^−1^) and water (18 cm^−3^ mol^−1^)^18^, the transformation results in a significant porosity in the newly formed calcite. Intermediate CaCO_3_ polymorphs (line vaterite) may also be formed during the transformation process, depending on pH and Mg concentration in the parent solution^11^. In this study only the precursor ACC and the product calcite formations were detected. The occurrence of the precursor and the product phases allowed us to develop a new sampling and measurement protocol for the preservation of possible ACC fraction and to determine the ACC oxygen isotope composition compared to calcite. Although the formation of intermediate phases like vaterite^11^ may affect the crystallization process, the isotope fractionation values would not be influenced^6^, hence the simplified transformation model (from a precursor phase of ACC to a product phase of calcite) can be used for the evaluation of the isotope data.

In general, during stalagmite formation, ACC-calcite transformation may occur in a closed or open system. If the ACC particles are on the stalagmite surface in contact with drip water during the transformation, then we can assume a fully open system, and hence the calcite attains equilibrium with the ambient water through a dissolution-reprecipitation process. If the precipitated ACC particles are covered by the subsequent carbonate layers (containing ACC and calcite), then the transformation may proceed in a closed system, through solid phase recrystallization. The possibility of ACC entrapment can be evaluated in the light of ACC particle sizes and the rate of carbonate deposition. If the thickness of the carbonate layer formed during the period of ACC transformation significantly exceeds the size of ACC particles, then the latter can be – at least partially – embedded. As ACC was detected in the freshly precipitated carbonate several days after the collection, we can assume that the transformation period is extended to days. Carbonate deposition rates can be estimated from the sizes (5 and 7 cm in height) and formation ages (~50 and ~210 years, respectively) of actively forming stalagmites (NU-1 and NU-2) at the sampling site^6^, yielding 0.3 to 1 mm yr^−1^ deposition rates, *i.e.*, deposition of 800 to 2700 nm thick carbonate layers per day. At such deposition rates 10–30 nm sized ACC particles can be trapped in the precipitated carbonate layer that precludes interaction with the ambient solution resulting in closed system ACC-calcite transformation. However, heterogeneous carbonate precipitation on the rugged stalagmite surface and temporal variation of the deposition rate produces spatially variable carbonate formation and hence can leave a part of the ACC uncovered. Hence, the most plausible assumption is that the ACC-calcite transformation during stalagmite formation at the study site takes place both in closed and open systems.

### Calcite-ACC oxygen isotope fractionation

In order to evaluate the influence of ACC formation on the use of stable oxygen isotope compositions in speleothem research, calcite-ACC oxygen isotope fractionation was determined by measuring the carbonate content of grids and silica wool placed directly beneath dripping water. Since the carbonate precipitated on the copper grids and silica wool is a mixture of calcite and ACC, the oxygen isotope composition of the calcite component should be determined along with the measurement of water composition. Hence, the stable isotope composition of the outer, ~3 mm thick part of a recently forming stalagmite (sample NU-2) collected at the Baradla Cave^6^ study site was also measured. Based on XRD and FTIR analyses, this stalagmite’s carbonate is composed of calcite (Figs 2 and 3). Since the stalagmite was actively growing at the time of collection, the oxygen isotope compositions of the stalagmite and the drip water can provide the calcite-water isotope fractionation value for the study site. The current fractionation value depends on temperature, pH conditions, growth rate, degassing and drip rates^19^,^20^,^21^,^22^, resulting in site-specific carbonate-water oxygen isotope fractionation relationships. Kinetic fractionation due to CO_2_ degassing and H_2_O evaporation related to strong ventilation can significantly affect the stable C and O isotope compositions of the precipitating carbonate^23^. If the transforming carbonate is in contact with an ambient solution whose composition is subject to change due to degassing and evaporation, then this solution may influence the O isotope composition of the newly formed calcite by resulting in elevated δ^18^O values. The potential influence of degassing and evaporation at the study site can be determined by conducting the so-called Hendy test^24^ on recently forming stalagmites. The results (presented and discussed in the Supplementary Material) indicate that, although carbon isotope composition naturally records the degassing effect (which is inherent in the carbonate precipitation process), it is not associated with systematic δ^18^O shifts. This observation does not imply that the carbonate precipitated in thermodynamic equilibrium with the drip water, but it shows that the degassing/evaporation effect may be considered negligible at the site and hence its influence on the carbonate–water system can be excluded.

The δ^18^O values of the NU-2 stalagmite calcite (23.4‰) and the drip water (−9.5‰) yields a site-specific fractionation value of 32.7 for the longer term (about 2 years) cave temperature (9.7 °C). Slight variations in cave temperature and drip water composition at the sampling site, however, may influence the oxygen isotope composition of the precipitating carbonate, hence current conditions prevailing during sample collection should be used for the evaluation. The theoretical oxygen isotope composition of the calcite component of the carbonate sample can be calculated from formation temperature and drip water composition, provided that the calcite-water oxygen isotope fractionation equation is known. Although a pioneering experimental study determined the temperature-fractionation relationship for the calcite-water system almost 50 years ago^25^, the definition of the true equilibrium calcite-water fractionation equation is still a hotly-debated topic^22^. Fractionation equations have been obtained using three different approaches: theoretical calculations, experimental analyses and empirical observations. Statistical mechanical calculations based on internal vibration frequencies yielded theoretical fractionation equations^26^,^27^ that may represent thermodynamic equilibrium. Experimental studies provided different equations^25^,^28^,^29^ depending on the experimental conditions (temperature range, precipitation mechanism, pH, growth rate)^19^,^22^, which may be distinctly different from those prevailing in natural environments. The third approach is empirical observations, i.e., measurement of calcite-water oxygen isotope fractionation for actively precipitating calcite in different environments (e.g. caves or thermal water occurrences)^30^,^31^,^32^,^33^. This approach bears most uncertainties as the precipitation conditions are not controlled, but the obtained equations contain all the unknown factors that govern calcite precipitation and may be most effectively applied as environment-specific relationships. Due to the large differences between theoretical, experimental and empirical equations, two empirical equations were selected as most representative for the studied caves’ environment. The formation conditions of the Devil’s Hole calcite (very low deposition rate in a closed environment) may be considered a closest-to-natural equilibrium^31^. Although the Coplen equation^30^ is based only on one fractionation value at 33.7 °C and a slope acquired from earlier studies, its applicability to extension to lower temperatures has been verified by later studies^22^,^34^, therefore the Coplen equation^30^ was selected to represent calcite-water equilibrium:


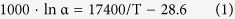


The other selected study^32^ is based on a comprehensive collection of real speleothem data, representing general cave environments that collectively contain all the natural factors which may have an effect (see above):


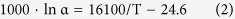


Since the α fractionation factor is the ratio of ^18^O/^16^O_cc_/^18^O/^16^O_water_, by definition:


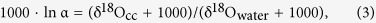


where “cc” stands for calcite.

Weighting equations (1) and (2) by a factor of 65 and 35%, respectively, would yield the observed calcite-water fractionation value of 32.7‰ at 9.7 °C. Current cave air temperatures (10.42, 9.34 and 9.27 °C) detected at the time of sample collections (Table 1) were substituted in equations (1) and (2) followed by a 65–35% weighing of the calculated 1000 · lnα values, as described above. Then the theoretical calcite oxygen isotope compositions were calculated using equation (3) and the measured water compositions (−9.1, −9.3 and −9.8‰), yielding δ^18^O_cc_ values of 23.8‰, 23.7‰ and 23.1‰ for the Baradla Cave grids, silica wool and the Pál-völgyi Cave silica wool samples, respectively. The composition of the ACC component in the sampled carbonate could be determined by simple mass balance calculation as follows, provided that the ACC amount in the precipitating carbonate is known.

The amount of the ACC component can be estimated on the basis of crystallinity data of actively forming stalagmites. Demény *et al*.^6^ presented XRD FWHM (full width at half maximum) data that showed a continuous decrease (from 0.16 to 0.12) from the stalagmite surface to the inner (older) layers for actively growing stalagmites collected in the close vicinity (within 50 m) of the sampling site of this study. FWHM values may depend on crystal domain size and lattice strain, but due to the absence of significant amounts of substituting ions or organic matter and physical deformation, the lattice strain effect can be excluded^6^. Attributing the observed peak broadening solely to domain size increase and using the classical Scherrer equation^35^ for the calculation, a domain size change from 55 nm to 70 nm would be obtained at the stalagmite surface and the innermost part of the NU-2 stalagmite, respectively. TEM analyses showed that the youngest layers contained nanocrystalline (<50 nm) calcite, whereas the oldest laminae were dominated by well crystallized carbonate^6^. As the presence of an amorphous carbonate fraction in the fresh precipitate has been proven by various methods, we suggest that the ACC fraction served as a precursor for the nanocrystalline calcite aggregates. Since ACC clusters are about 10–40 nm in size, a similar crystal size can be assumed for the initial nanocrystalline calcite. The 70 nm size can be attributed entirely to well crystallized calcite (S_cc_), whereas the 55 nm average size (S_avg_) at the stalagmite surface would be determined by the size and the amount of ACC-derived nanocrystalline calcite (S_ACC_ and X_ACC_, respectively) according to a simple mass balance equation:


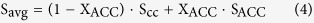


The size estimations (S_cc_ = 70 nm, S_avg_ = 55 nm, S_ACC_ = 10–40 nm) determine the amount of the nanocrystalline fraction (X_ACC_) that ranges from 0.25 to 0.5. Since a part of this size fraction can be precipitated originally as nanocrystalline calcite, the 25–50% range represents an upper limit to the ACC amount estimation. The ACC amounts and the theoretical calcite composition (δ^18^O_cc_) can be used to calculate the isotopic composition of the ACC fraction (δ^18^O_ACC_) using the equation


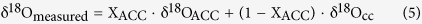


For the Baradla grid and silica wool samples, the calcite end-member composition calculations (using the calcite-water fractionation relationships and measured cave temperatures as described above) yielded 23.8 and 23.7‰, whereas the Pál-völgyi Cave site yielded 23.1‰. Substituting the 25–50% ACC amount in the carbonate mixture (X_ACC_ = 0.25 to 0.5) in equation (5), an average calcite-ACC δ^18^O difference of 2.4 ± 0.8‰ is obtained. As a part of the nanocrystalline fraction may also be precipitated originally as calcite, the X_ACC_ values are maximum estimations, and lower X_ACC_ values would yield larger calcite-ACC δ^18^O differences. An additional source of uncertainty derives from the partial transformation of ACC to calcite during the storage period between sample collection and isotope analyses. Although the low temperature in the refrigerator (2–5 °C) precludes solid-phase carbonate-water oxygen isotope exchange, structural reorganization during calcitization may also be associated with isotope exchange between the newly formed calcite and ambient water (containing the released hydration water and the ambient H_2_O vapor). At such low temperatures, calcite-water isotope fractionation is elevated relative to the studied cave’s temperature (~10 °C)^25^, leading to ^18^O-enrichment in the calcite. The presence of such newly formed calcite would mean that the original carbonate sample should have had lower δ^18^O value than measured, and consequently the calculated calcite-ACC difference should be larger. Transformation of ACC to calcite during sample storage also leads to the assumption that the calcite-ACC difference (~2.4‰) represents a lower limit.

Uncertainties in the estimation of ACC amount is a major weakness in the fractionation calculation, hence the verification of calcite-ACC fractionation estimation requires independent information provided either by experimental studies or by natural analogues. The experimental determination of ACC-water oxygen isotope fractionation representative for speleothem formation is challenging because (i) ACC rapidly transforms to calcite during the preparation and (ii) laboratory ACC synthesis requires physical and chemical conditions distinctly different from those found in a cave environment. Available estimations of δ^18^O differences between crystalline and amorphous carbonates formed in natural environments suggest that the crystalline carbonate is several ‰ more enriched in ^18^O than its amorphous counterpart (dolomite^7^, aragonite^8^, Mg-calcite^9^). These estimations are in agreement with the result of the present study, which suggests a >2.4‰ calcite-ACC δ^18^O difference at ~10 °C.

### Implications for paleoclimate research

As a general rule, speleothems with well developed lamination and compact texture (non-porous texture with columnar or fibrous crystals) are selected for paleoclimate analyses^36^,^37^, as they are less prone to diagenetic alteration. The formation of a porous texture and late-stage re-crystallization both enhance post-depositional exchange with ambient solutions, leading to changes in the original stable isotope and trace element compositions^2^,^3^,^4^ during open system alteration. The formation of relatively ^18^O-depleted ACC and its subsequent transformation into calcite can also exercise an especially significant effect in fluid inclusion research, inducing oxygen isotope re-equilibration between the host carbonate and water trapped in fluid inclusions^6^ if the mineralogical transformation took place in a closed system. In general, primary fluid inclusions are thought to derive from entrapment of drip water solution during carbonate precipitation. However, in the present case hydration water released during ACC transformation to calcite may also be trapped, contributing to the inclusion-hosted water content. As pointed out above, fast deposition of primarily precipitated calcite may cover and enclose a part of ACC that is later transformed within the calcite host in a closed system. The fluid inclusions within or in contact with ACC would approach the calcite-water oxygen isotope equilibrium during the transformation and subsequent re-crystallization^6^. As the amount of water is negligible compared to the host carbonate, the inclusion water composition would be forced to shift to more negative δ^18^O values in order to achieve the larger calcite-water fractionation relative to the ACC-water value. As discussed by Demény *et al*.^6^, speleothems from warm/tropical regions frequently yield meaningful δ^18^O values for inclusion-hosted water, whereas the oxygen isotope compositions of inclusion-hosted water obtained for samples from cold/temperate regions are generally not appropriate for paleotemperature calculations^6^. A potential reason for this discrepancy is formation of ACC in cold/temperate caves whose transition to calcite induces δ^18^O shifts in the inclusion water^6^. The present study provides direct evidence for relatively ^18^O-depleted ACC formation in caves at about 10 °C. Since the δ^18^O value of inclusion-hosted water may carry significant paleoclimatic/paleohydrological information, it is important to note that its use is limited by the cave environment.

### Demand for future studies

A number of experimental studies have shown that the formation and stability of ACC may be influenced by the physical parameters of the ambient environment and the chemical compositions of the parent solutions. In natural cave environments the most important factors might be the cave temperature, drip water pH, as well as concentrations of Mg, SO_4_^2−^ and organic compounds in the solution^10^,^11^,^15^,^38^,^39^,^40^. A comprehensive study is suggested to cover several cave environments with different temperatures, ventilation degree, soil characteristics, drip water chemistry and carbonate growth rates in order to determine the exact factors governing ACC formation. The transition from ACC to calcite has been shown to take place in several steps with intermediate hydration states and mineral phases like vaterite^12^,^13^,^14^,^17^,^41^. Investigations on the ACC-calcite transition and its governing factors require monitoring of mineralogical changes at high temporal resolution. Additionally to the inorganic factors, the role of microbial activity should also be investigated. Amorphous carbonates are ubiquitously secreted by living organisms in sedimentary environments^42^, hence microbial mediated carbonate precipitation is also a potentially important process in ACC formation, whose exploration requires systematic biological/biochemical investigations.

## Conclusions

The formation of ACC was detected in carbonate samples precipitated from drip water in cave environments monitored for temperature, drip water pH, conductivity and stable isotope compositions. In order to capture and analyse very small amounts of carbonate without further sample concentration, Cu grids used for TEM and quartz wool were placed under drip water for times ranging between 24 hours and two weeks at two cave sites (the Baradla and Pál-völgyi Caves, Hungary). Globular forms of carbonate were observed on the copper grids using SEM; these then transformed into porous carbonate after several weeks. FTIR spectra, X-Ray powder diffraction and TEM analyses of freshly precipitated carbonates collected on TEM Cu grids showed the characteristic features reported for synthetic ACC precipitates. ACC occurred as films containing clusters 10–30 nm in size and as particles 10–40 nm in size. It may be assumed that this material is capable of serving as a precursor of nanocrystalline calcite found in the outermost layer of recently formed speleothems^6^.

The stable oxygen isotope compositions of the freshly precipitating carbonate, stalagmite calcite and host drip water were used to infer oxygen isotope fractionation between calcite and ACC. The calcite-ACC isotope fractionation estimation obtained in this study (>2.4 ± 0.8‰) is in agreement with those obtained from the data contained in earlier publications^8^,^9^. These observations further support the earlier hypothesis^6^ that closed-system transformation of ACC into calcite induces oxygen isotope changes in the fluid inclusion water of stalagmites as the carbonate-water system approaches calcite-water equilibrium fractionation. The further implication of our results is that ACC transformation into nanocrystalline carbonate and the recrystallization of the latter to well crystallized calcite increases the chance of late-stage interactions with infiltrating solutions. This open-system carbonate-water interaction during re-crystallization can lead to – at least a partial – loss of primary geochemical signals, affecting the speleothems’ sensitivity to diagenetic alteration and their applicability to paleoclimate research. As temperature rises, the extent of ACC precipitation decreases^7^, hence ACC formation may be of particular importance in cold-temperate caves. Besides cave monitoring, the mineralogical analysis of freshly precipitating carbonate at the location of speleothem collection is suggested in order to determine the potential influence of ACC formation in the cave site under study.

## Analytical Methods

Fresh carbonate precipitate was collected in two caves, the Baradla Cave in the northeastern part of Hungary, and the Pál-völgyi Cave in Budapest, central Hungary. Temperature, pH, conductivity, and CO_2_ content in cave air were monitored at both locations and water samples were collected. Cave air and water temperatures were measured using a *GMH-3710* type Pt-100 thermometer digital puncture (range from −199.99 to +199.99 °C, resolution: 0.01 °C) with a precision of ±0.03 °C. CO_2_ contents in cave air at 150 cm from the ground were determined using different NDIR instruments for low (*TESTO-535,* range 0 to 10 000 ppm, resolution: 1 ppm) and high (*ANALOX ASPIDA,* range 0 to 10 vol%, resolution: 0.01 vol%) levels. Electrical conductivity and pH values of dripping water and cave river waters were measured within 1 minute after sampling using a *COMBO PH & EC* instrument (EC range 0 to 3999 μS/cm, resolution: 1 μS/cm, pH range 0.00 to 14.00, resolution: 0.01) with precisions of 2% and 0.05, respectively. Plastic funnels holding Cu grids and silica wool over a filter were placed under the dripwater at both caves. Grids and silica wool samples were collected from 24 hours to two weeks. The samples were immediately placed in a cold box and transported to the laboratory, where they were stored in a refrigerator at 2–5 °C. According to literature data, low temperature can help stabilizing ACC^38^.

X-ray powder diffraction (XRD) analysis of stalagmite carbonate (sample NU-2/1) was performed using a Philips PW-1730 diffractometer (PW-1820/00 goniometer) equipped with a graphite monochromator using Cu-Kα radiation at 45 kV and 35 mA with 1° divergence slit and 1° receiving slit. Scanning rate was 0.05° 2Θ per minute from 3° to 70°. Micro-focus X-ray diffraction measurements were made by using D/MAX RAPIDII type diffractometer and RINTRAPID software at the Institute for Geological és Geochemical Research (IGGR), Budapest. Measurement conditions were 50 kV accelerating voltage, 0.6 mA current, using a 800 micrometer beam collimator. The analysed Cu grid (grid #2) was held in a fixed position during measurement. The 2DP software was applied to convert the Debye-rings into 2theta intensity values with 0.01 steps.

FT-IR (Fourier-transformation infrared) analyses were carried out using a Bruker Vertex 70 IR spectrometer equipped with a Hyperion 2000 microscope with video-controlled visualization and automatized sample stage operated by OPUS 7.2 software, at the IGGR, Budapest. The measurements were performed in transmittance mode using a 15X objective. For each sample 32 scans were recorded in the 4000–400 cm^−1^ spectral range with a resolution of 4 cm^−1^.

TEM studies of the samples collected on the Cu grids were carried out in a Philips CM20 (accelerating voltage: 200 kV, LaB6-filament) type transmission electron microscopes at Institute of Technical Physics and Materials Science of the Centre for Energy Research, Budapest. Bright-field images and selected area diffraction (SAED) patterns were recorded on CCD camera (Morgagni microscope) and image plates (Philips microscope). Radial distributions of intensities were generated from SAED patterns following the method described by Lábár^43^ and using the ProcessDiffraction software. Scanning electron microscope images were acquired by using a ZEISS EVO 40XVP Scanning Electron Microscope (accelerating voltage: 20 kV, W-filament, working distance 10 mm) at the Institute of Materials and Environmental Chemistry, Budapest.

Carbonate-bearing Cu grids and silica wool samples were divided to several sub-samples and placed in borosilicate exetainers, flushed with helium and reacted with pure H_3_PO_4_ at 70 °C in an automated GASBENCH II preparation unit^44^ attached to a Thermo Finnigan delta plus XP mass spectrometer at the IGGR, Budapest. Due to the accidental carbonate precipitation the CO_2_ yields were variable. The sample signals were monitored and samples below 50 μg and above 200 μg were excluded in order to match the standards’ signal (for about 150 μg calcite) and to avoid linearity related isotope shifts. Stable carbon and oxygen isotope compositions were determined on the evolved CO_2_ and are expressed as δ^13^C and δ^18^O in ‰ relative to V-PDB and V-SMOW, respectively. Precision and accuracy of stable carbon and oxygen isotope compositions were tested by analysing international standards that yielded the following compositions. NBS-18: δ^13^C = −5.03 ± 0.06‰, δ^18^O = −23.29 ± 0.07‰ (n = 16); NBS-19: δ^13^C = −1.92 ± 0.07‰, δ^18^O = −2.22 ± 0.04‰ (n = 16); LSVEC: δ^13^C = −46.60 ± 0.16‰, δ^18^O = −26.60 ± 0.08‰ (n =17), all of the values within 0.1‰ from the certified compositions reported by the International Atomic Energy Agency. Stable oxygen isotope compositions of drip water samples (δ^18^Ow values relative to V-SMOW) were determined using a liquid water isotope analyser (LWIA) manufactured by Los Gatos Research Ltd, model LWIA-24d with an analytical precision of 0.1‰^6^.

## Additional Information

**How to cite this article:** Demény, A. *et al*. Formation of amorphous calcium carbonate in caves and its implications for speleothem research. *Sci. Rep.* **6**, 39602; doi: 10.1038/srep39602 (2016).

**Publisher's note:** Springer Nature remains neutral with regard to jurisdictional claims in published maps and institutional affiliations.

## Supplementary Material

Supplementary Information

## Figures and Tables

**Table 1:** Air and water temperatures, CO_2_ contents in cave air, pH and conductivity of dripwater, and stable isotope compositions (as δ^13^C and δ^18^O values relative to V-PDB and V-SMOW, respectively) of carbonate (“carb”) deposited on copper grids and silica wool and dripwater samples (“w”) at two cave sites (Baradla and Pál-Völgyi Caves, Hungary).

Date	T(air) °C	T(water) °C	CO_2_ ppm	pH	Conductivity (μS/cm)	δ^13^Ccarb ‰	δ^18^Ocarb ‰	δ^18^Ow ‰
**Baradla Cave**								
2015.11.13	9.54	9.53	5700	8.03	840			
2015.11.18	9.52	9.69	3100	8.09	881	**grids:**		
2015.12.01	10.42	10.77	2300	7.36	743	−11.26	22.91	−9.1
2015.12.02	10.62	10.75	3700	7.48	765	**silica wool:**		
2015.12.19	9.34	9.37	3300	8.22	767	−11.63	23.08	−9.3
2016.01.02	9.42	9.61	2400	8.03	815			
**Pál-völgyi Cave**								
2015.11.30	10.59	10.57	800	8.03	2187	**silica wool:**		
2015.12.15	9.27	10.17	643	8.21	2394	−7.83	22.14	−9.8
**Baradla Cave, NU-2 stalagmite, upper 3 mm**						−9.9(±0.3)	23.4(±0.3)	
**Baradla Cave, dripwater compositions**								
	**Na**	**Mg**	**Al**	**Si**	**P**	**Ca**	**Sr**	**Ba**
2015.11.13	1188.42	2541.43	2.89	3309.60	62.09	34999.64	73.50	51.18
2015.12.01	1034.37	1800.07	2.23	3004.24	50.46	38596.21	70.87	18.67
2015.12.19	961.32	1690.98	2.96	2864.10	45.80	40339.43	67.43	18.71

Chemical compositions of dripwater samples are in ppm (see analytical methods in Demény *et al*.)^6^.
