# Supplementary material for: Formation of amorphous calcium carbonate in caves and its implications for speleothem research
Source: Sci Rep. 2016 Dec 22;6:39602. doi: 10.1038/srep39602 (PMC5177889; doi:10.1038/srep39602)
Supplement: Supplementary Information [file srep39602-s1.pdf]

Formation of amorphous calcium carbonate in caves and its implications for speleothem research

Supplementary Material

Attila Demény[1*], Péter Németh[2], György Czuppon[1], Szabolcs Leél-Őssy[3], Máté Szabó[1], Katalin Judik[1], Tibor Németh[1], József Stieber[4]

[1] Institute for Geological and Geochemical Research, RCAES, Hungarian Academy of Sciences, Budaörsi út 45, Budapest, H-1112, Hungary

[2] Institute of Materials and Environmental Chemistry, Research Centre for Natural Sciences, Hungarian Academy of Sciences, Magyar tudósok körútja 2., Budapest, H- 1117, Hungary

[3] Department of Physical and Applied Geology, Eötvös Loránd University, Pázmány Péter sétány. 1/C, Budapest, H-1117, Hungary

[4] Stieber Environmental Ltd., Nyerges u. 6., Budapest, H-1181, Hungary

[*] Correspondence to demeny@geochem.hu

Hendy test analyses of actively growing stalagmites at the Baradla cave study site

In a well ventilated environment the dripwater layer flowing down on the stalagmite's surface is subjected to coupled CO_2 degassing and H_2O evaporation that together drive the precipitating carbonate's C and O isotope compositions in a positive direction due to kinetic fractionations[23]. Measuring $\delta^{13}C$ and $\delta^{18}O$ values along the same growth lamina moving away from the growth axis (“Hendy test”) can detect the effect of kinetic fractionation[24]. Carbonate precipitation is naturally related to CO_2 degassing as it results in carbonate oversaturation in the solution, hence $\delta^{13}C$ increase along the growth lamina is expected. Strong ventilation would enhance not only CO_2 degassing (leading to fast carbonate precipitation and hence to kinetic fractionation), but also water evaporation that causes ^{18}O-enrichment in the solution and in the precipitating carbonate, resulting in positive $\delta^{18}O$-$\delta^{13}C$ correlation. As a consequence, plotting the $\delta^{13}C$ and $\delta^{18}O$ values as a function of distance from the growth axis may be used to detect the kinetic fractionations.

Two actively forming stalagmites[6] with very different growth rates were collected at the study site (within 50 m from the fresh carbonate sampling location). The stalagmites were cut using a diamond saw and polished. Both stalagmites are characterized by well visible lamination[6] consisting annual laminae that are possible to follow and sample from the top to the the flanks. Three laminae from both stalagmites were sampled and analysed, the results are shown in Fig. S1. The figure shows that no systematic positive $\delta^{18}O$ shift is associated with $\delta^{13}C$ changes along the growth laminae, leading to the conclusion that ventilation-related kinetic fractionation is not characteristic for the study site.

See main text for references.

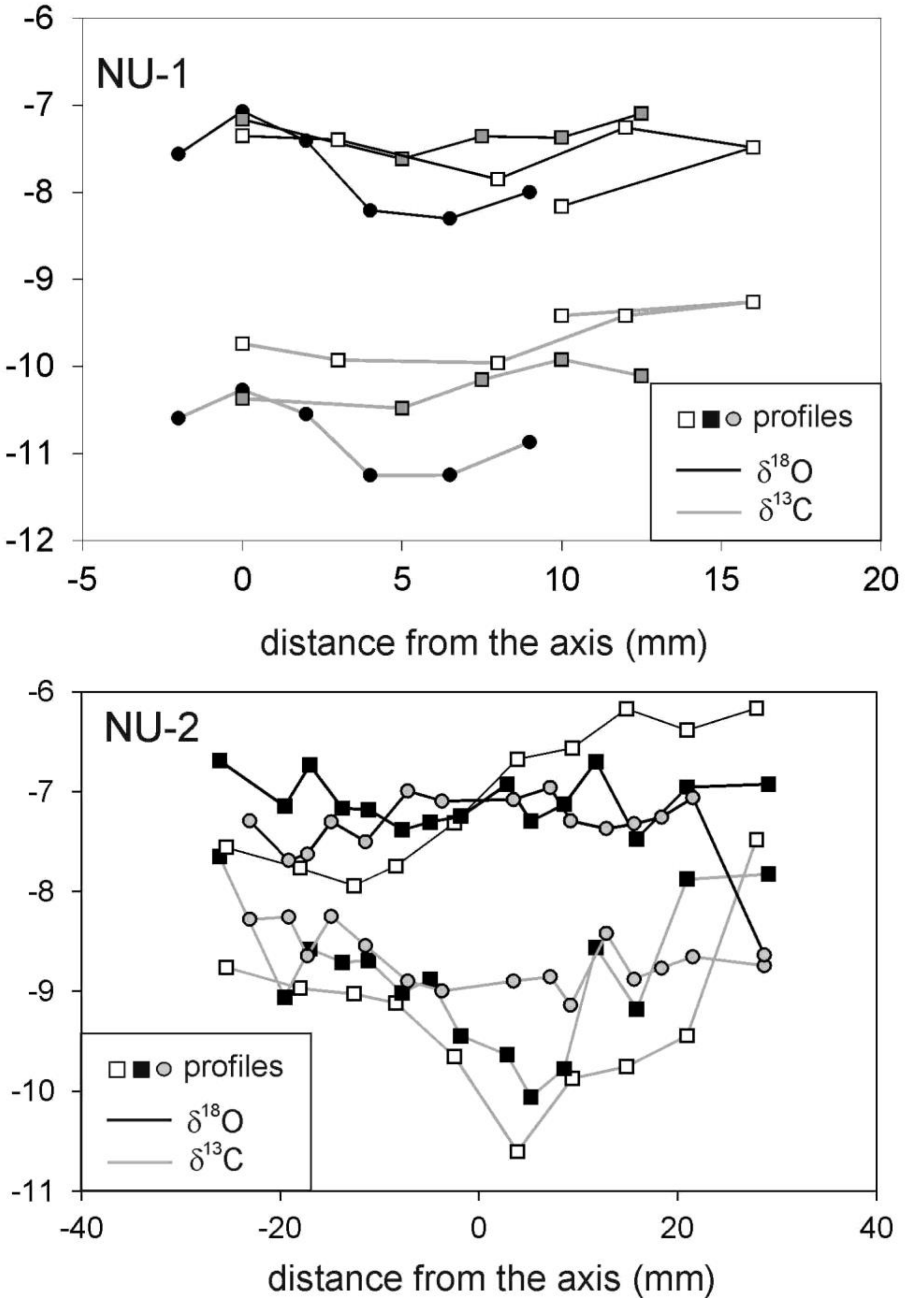

Figure S1. Stable carbon and oxygen isotope compositions (in ‰ relative to V-PDB) as a function of distance from the growth axes along growth laminae of the NU-1 and NU-2 stalagmites.
